# Operational developments at the *Journal of the Medical Library Association*: improved production timelines, new policy introduction, and senior editorial team updates

**DOI:** 10.5195/jmla.2025.2289

**Published:** 2025-07-01

**Authors:** Jill T. Boruff, Michelle Kraft

**Affiliations:** 1 jill.boruff@mcgill.ca, Co-Lead Editor, *Journal of the Medical Library Association*, Associate Librarian, Schulich Library of Physical Sciences, Life Sciences, and Engineering, McGill University, Montreal, QC, Canada; 2 kraftm@ccf.org, Co-Lead Editor, *Journal of the Medical Library Association*, Medical Library Director, Cleveland Clinic Foundation, Cleveland, OH, United States

## Abstract

In our editorial in the January/April 2023 issue of the *Journal of the Medical Library Association* (*JMLA*), we spoke of the challenges we faced when we took on the co-lead editor roles. At the end of that editorial, we stated our intention to get the publishing schedule back on track and to finally tackle other projects. And while it took us some time to report it, we are pleased to share that, in the publication year of 2024, *JMLA* resumed its regular quarterly publishing schedule.

## PRODUCTION TIMELINES

In our editorial in the January/April 2023 issue of *JMLA*, we spoke of the challenges we faced when we took on the co-lead editor roles. At the end of that editorial, we stated our intention to get the publishing schedule back on track and to finally tackle other projects. And while it took us some time to report it, we are pleased to share that, in the publication year of 2024, *JMLA* resumed its regular quarterly publishing schedule.

We also improved the time that it takes for a manuscript to work its way through the publication process. The following data from our peer reviewed publication categories of Knowledge Synthesis, Original Investigation, and Case Report demonstrate this shortened timeline. In 2024, 80% of submissions received notification of acceptance in 234 days or notification of decline in 23 days. The 234 days represents the time it has taken for a manuscript to go through one or more rounds of peer review and various rounds of revisions, before being accepted for publication. Unfortunately, we do not have a data point to report the average amount of time it takes for a manuscript authors to hear back from the first round of peer review, but as we reported in our April 2024 editorial, we strive to have this response back within six weeks of submission. This metric of 234 days-to-accept for 80% of our submissions in 2024 represents a significant improvement from 2022, where 80% of our submissions waited 346 days, or nearly a year, for acceptance.

We are also pleased to report that we have had a steady number of manuscript submissions, with 142 in 2022, 152 in 2023, and 165 in 2024. From these submissions, we have accepted 29, 32, and 32 peer-reviewed manuscripts respectively. We look forward to reading your future submissions!

As editors we are always seeking individuals with a diverse set of knowledge and skills in health sciences libraries to serve as peer reviewers. It is important to have a deep pool of reviewers in many subjects and areas of expertise, especially in new and emerging trends, so that authors and readers can fully benefit from the peer review process. *JMLA*'s website has links to several resources on the peer review process and how to provide good peer review. If you are interested in serving as a peer reviewer please contact us at jmla@journals.pitt.edu.

As generative artificial intelligence (GenAI) continues to have an impact on the publishing and research landscape, *JMLA* is proud to be one of the first library journals to have established a policy on the use of GenAI. We continue to monitor the evolution of AI so that we can adjust our policy accordingly. We look forward to publishing articles on GenAI and its impact in libraries and research methodology.

## NAME CHANGE POLICY

One of the projects that we were finally able to implement was an official name change policy for JMLA. Thanks to the hard work of the JMLA Equity Group, who drafted the policy with input from the senior editorial team, we now have a clear procedure for any JMLA author who would like to request a name change. Name changes are available to authors upon request with no explanation or legal documentation required. This policy does not apply to author name misspellings due to copy-editing errors or affiliation changes. The lead editor will work with the production editor to update all digitally published content, metadata, and associated records under our control to reflect the requested name change, and we will not issue a notice of correction for the name change. For more information about this policy, please see our name change policy webpage.

The *JMLA* editorial board is committed to collaborating with other groups to advocate for systemic changes to ensure author name changes are fully supported in academic publishing. To that end, we strongly encourage all contributors to *JMLA* to utilize open systems like ORCiD which allow authors, researchers, and scholars to create profiles with associated unique, persistent identifiers independent of their legal names and/or institutional affiliations.

## SENIOR EDITORIAL TEAM UPDATES

Kathleen Amos has stepped down from her role as Associate Editor, a position she has held for nearly a decade. After completing a two-year term within the National Library of Medicine's Associate Fellow program, Kathleen joined the Public Health Foundation (PHF) as a librarian through the Sewell Learning Partnership program. While in this role, Kathleen published her first article within *JMLA*, exploring plagiarism and duplicate publications within biomedical literature. Although Kathleen's portfolio at PHF quickly grew to includes responsibilities beyond health sciences libraries, her commitment to leading change within the scholarly publishing ecosystem has never wavered. Kathleen leaves *JMLA* as our longest tenured current editorial member; her generosity, humor, and publishing insight will be dearly missed by her colleagues across the journal.

We are delighted to welcome Robin Parker, MLIS, PhD, to the senior editorial team as Assistant Editor, after serving on the *JMLA* Editorial Board from 2021 to 2024 and as a peer reviewer for many years. Robin has significant experience with qualitative research, having completed an interdisciplinary PhD with a digital ethnographic study using sociomaterialism as a framework to examine how health librarians teach knowledge synthesis methods in online environments. Her dissertation described the invisible labour and specialized expertise of librarians supporting learners through the relationships amongst research and communication technologies, online pedagogies, and review methodologies. Robin works as the Evidence Synthesis Librarian and library liaison supporting undergraduate, graduate, and postgraduate Medicine programs at the W.K. Kellogg Health Sciences Library at Dalhousie University in Nova Scotia, Canada. In addition to her interests in using qualitative research methods to understand the role of context in health libraries and health professions education, Robin has extensive experience with review methodologies and systematic searching. She has been involved with the Cochrane Collaboration, including as a Cochrane author for over 15 years. Robin looks forward to supporting authors interested in publishing their qualitative and mixed methods research with JMLA.

Finally, we are pleased to announce our recent re-appointment as the Co-Lead Editors of JMLA. It has been an honor to serve, and we look forward to being able to implement more of our ideas over the next three years.

